# Analysis of passive cardiac constitutive laws for parameter estimation using 3D tagged MRI

**DOI:** 10.1007/s10237-014-0638-9

**Published:** 2014-12-16

**Authors:** Myrianthi Hadjicharalambous, Radomir Chabiniok, Liya Asner, Eva Sammut, James Wong, Gerald Carr-White, Jack Lee, Reza Razavi, Nicolas Smith, David Nordsletten

**Affiliations:** 1 Division of Imaging Sciences and Biomedical Engineering, King’s College London, 4th Floor, Lambeth Wing St. Thomas’ Hospital, Westminster Bridge Road, London, SE1 7EH UK; 2 Faculty of Engineering, The University of Auckland, ENGINEERING BLOCK 1, Level 5, Room 401-509, 20 SYMONDS ST, Auckland, 1010 New Zealand

**Keywords:** Cardiac mechanics, Constitutive laws, Parameter estimation, 3D tagged MRI, Patient-specific modeling, Practical identifiability

## Abstract

An unresolved issue in patient-specific models of cardiac mechanics is the choice of an appropriate constitutive law, able to accurately capture the passive behavior of the myocardium, while still having uniquely identifiable parameters tunable from available clinical data. In this paper, we aim to facilitate this choice by examining the practical identifiability and model fidelity of constitutive laws often used in cardiac mechanics. Our analysis focuses on the use of novel 3D tagged MRI, providing detailed displacement information in three dimensions. The practical identifiability of each law is examined by generating synthetic 3D tags from in silico simulations, allowing mapping of the objective function landscape over parameter space and comparison of minimizing parameter values with original ground truth values. Model fidelity was tested by comparing these laws with the more complex transversely isotropic Guccione law, by characterizing their passive end-diastolic pressure–volume relation behavior, as well as by considering the in vivo case of a healthy volunteer. These results show that a reduced form of the Holzapfel–Ogden law provides the best balance between identifiability and model fidelity across the tests considered.

## Introduction

Cardiac imaging provides a powerful tool for assessing cardiac function and pathology, offering valuable insight into the kinematics and tissue characteristics of the heart. Its joint use with multiscale mathematical models of the heart (Costa et al. 2001; Guccione et al. 1995; Nash and Hunter 2000; Holzapfel and Ogden 2009; Chapelle et al. 2011) enables the quantification of model constitutive parameters, which can be used as clinical biomarkers of disease (Wang et al. 2009; Xi et al. 2011b; Sermesant et al. 2006; Chabiniok et al. 2012; Imperiale et al. 2011). As a result, there is a strong need for reliable parameter estimates, an issue which depends on both the underlying cardiac constitutive model as well as the available clinical data.

A variety of noninvasive cardiac imaging techniques have been developed, which offer a powerful set of tools for personalization of mechanical parameters. Imaging methods such as echocardiography, computed tomography (CT), cardiac magnetic resonance imaging (MRI) have been successfully employed to accurately capture epicardial and endocardial motion, thus providing important bulk metrics such as ejection fraction and cavity volumes. In addition, the development of speckle tracking echocardiography (Meunier 1998; Craene et al. 2012) and cardiac MR tagging [SPAtial Modulation of Magnetization, SPAMM (Zerhouni et al. 1988; Axel and Dougherty 1989; Reichek 1999)] has enabled the quantification of regional cardiac motion in vivo (Young et al. 1995; Osman et al. 1999; Arts et al. 2010), by revealing local characteristics such as wall thickening, torsion and shear effects. The translation from 2D to 3D tagging techniques (Rutz et al. 2008) has enabled a direct extraction of the full 3D displacement field in the myocardium, leading to simultaneous quantification of radial, circumferential and longitudinal motion (Shi et al. 2012; Pan et al. 2005). This accurate “whole-ventricle” 3D deformation field creates an ideal setting for estimation of model-based parameters using tissue displacement observations.

The wealth and quality of information on myocardial motion has enabled patient-specific applications, where passive cardiac constitutive laws of varying complexity have been employed, ranging from simplified isotropic (Cheng et al. 2005) and transversely isotropic (Guccione et al. 1991) laws to orthotropic models accounting for the fiber anisotropy of the tissue (Holzapfel and Ogden 2009; Costa et al. 2001; Nash and Hunter 2000). However, as the model complexity and number of parameters increase to better approximate the tissue’s complex behavior, estimating model parameters uniquely and accurately becomes an increasingly challenging task (Xi et al. 2011b). This raises the important question of *structural identifiability* for the various constitutive laws, i.e., whether it is possible to uniquely determine parameter values, given infinite well-defined noise-free data (Chis et al. 2011; Raue et al. 2009). Structural identifiability—a property of the model itself which does not depend on the available data—can be compromised by coupling between model parameters as in the case of the Guccione model (Wang et al. 2009; Xi et al. 2011a, b; Augenstein et al. 2005) and nonlinear dependence of the model on the parameters. Lack of structural identifiability hinders the ability of any data assimilation method—mainly categorized into variational (Sun et al. 2009; Augenstein et al. 2005; Wang et al. 2009; Sermesant et al. 2006) and sequential (Moireau et al. 2008, 2009; Chabiniok et al. 2012; Xi et al. 2011b; Wong et al. 2007; Liu and Shi 2009)—to accurately estimate parameter values.

In a clinical scenario, the estimation process is further compromised by limited data and measurement noise, leading to the issue of * practical identifiability*, i.e., whether we can determine unique parameter estimates given the limited amount and quality of data (Saccomani 2013). Absence of structural or practical identifiability in a cardiac law given a set of data leads to unreliable parameters, which can no longer provide clinically relevant information. The choice of an appropriate cardiac constitutive law should therefore balance the need for *model fidelity*, i.e., the ability of the model to accurately represent cardiac function, with the requirement for reliable identifiable parameters.

**Fig. 1:**
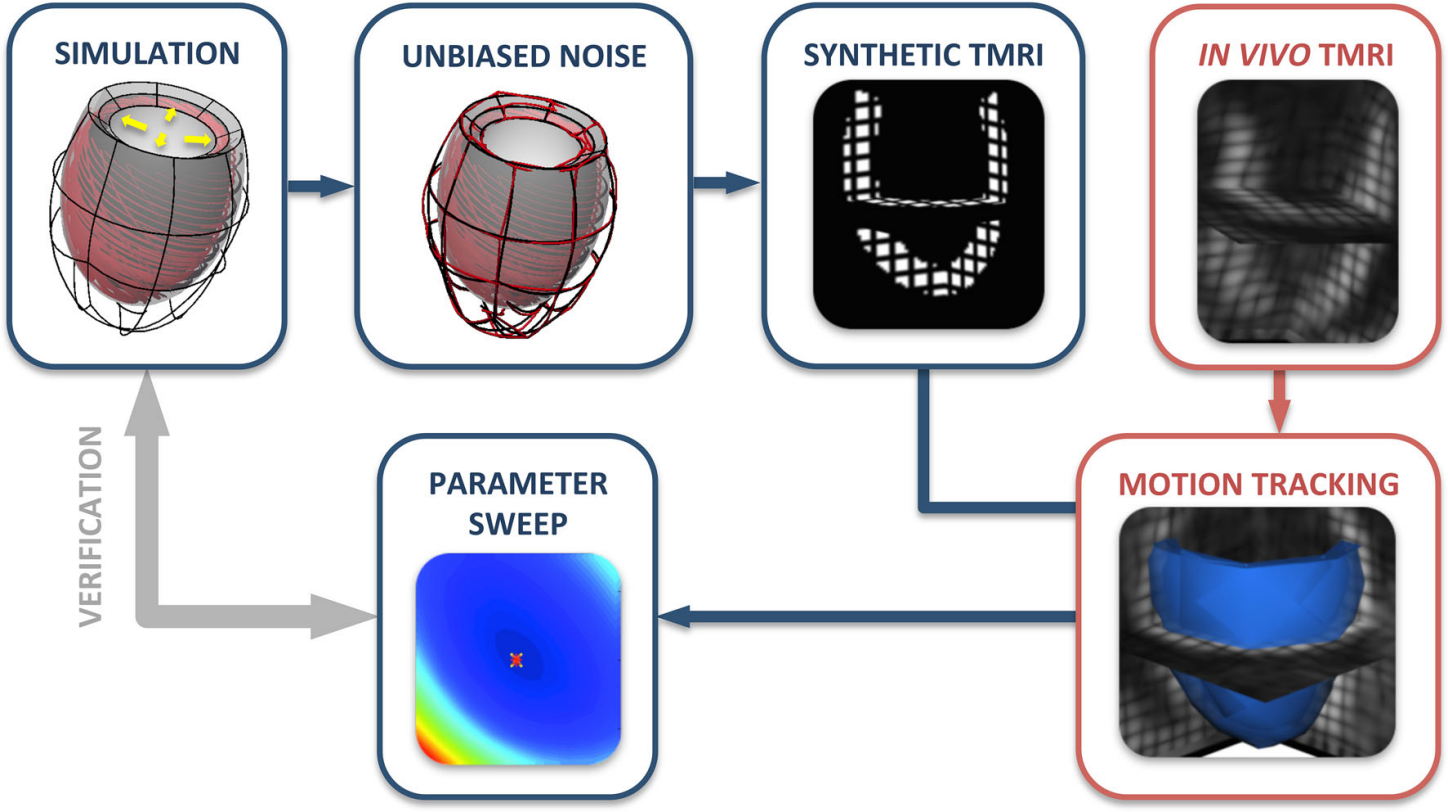
Workflow followed for the study of practical identifiability using 3D tags. The in silico testing protocol is presented in *blue*, while in *red* is the pipeline followed in the in vivo case

In this work, we aim to assist the choice of an appropriate constitutive law when the available data are 3D tagged MRI, by examining the practical identifiability and model fidelity of different cardiac mechanics models. Specifically, we look to compare progressively complex models to assess their capacity to both represent cardiac motion and be used reliably for parameterization. In order to gain insight into the parameter estimation process for these models, we investigate the behavior of a minimization criterion ($\mathcal {J}$) over the parameter space. This is first tested using synthetic 3D tags extracted from in silico simulations and performing parameter sweeps to obtain the parameter estimates. Following the work-flow described in Fig. 1, we characterize $\mathcal {J}$ and assess the error between estimated and actual parameters. The various models are further compared with respect to their ability to represent physiological cardiac deformation (model fidelity) and end-diastolic pressure–volume relation (EDPVR), in order to identify a constitutive law that would balance between practical identifiability and adequate representation of cardiac behavior. Our conclusions are then verified in an in vivo case, validating our in silico framework for characterizing practical identifiability using 3D tags.

Below, we expand on our approach to investigate practical identifiability and how it is influenced by the choice of constitutive law. The process for characterizing practical identifiability for each one of the considered models is reviewed in Sect. 2 and employed for in silico tests of diastolic filling using an idealized left ventricle (Sect. 3). The study is then extended to an in vivo case of a healthy volunteer, enabling the characterization of practical identifiability and model fidelity in a real-world scenario.

## Methods

In this section, we describe the process followed in this work in order to assess the practical identifiability of various laws, focusing on the creation of synthetic tags, the motion extraction algorithm used, and the parameter sweeps performed (Sect. 2.1). We then present the cardiac model of LV diastolic filling used, as well as the various cardiac constitutive laws considered (Sect. 2.2). Finally, we review a general theoretical framework for the inverse problem of parameter estimation using 3D tags (Sect. 2.3), focusing on the concepts of structural and practical identifiability, and the factors that influence them (observations, constitutive laws, objective function).

### In silico tagging and assessment protocol

**Fig. 2:**
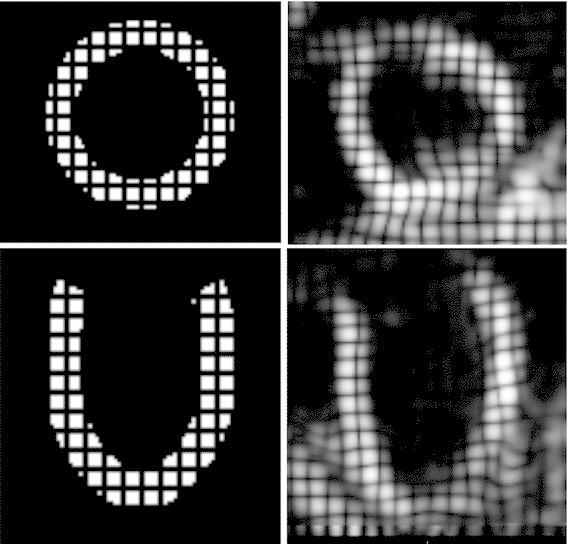
Comparison between synthetic (*left*) and real (*right*) 3D tags at beginning of diastole, short-axis view on *top figures*, long-axis view on *bottom figures*

A primary goal of this study was to assess the potential of using 3D tagged MRI in parameter estimation applications. Even though 3D tagged MRI offers a rich dataset for parametrization, the process may be compromised by low-resolution or noisy data and error introduced during the motion-tracking procedure. In order to investigate this issue, we have created synthetic 3D tagged images directly from simulation results. Within this controlled environment, the actual parameters of the heart model are known, allowing for an assessment of the error between actual and estimated parameters. Further, as the synthetic tags approximate real 3D tagged images (see Fig. 2), within this framework, we can quantify the error associated with various aspects of 3D tags such as resolution, number of tag lines, noise in the data, and error introduced by the tracking algorithm.

Initially, we ran a simulation of LV diastolic filling, choosing parameters that produce a physiological end-diastolic volume. Synthetic 3D tags were then generated from the resulting deformation, and the myocardial motion was extracted and propagated on a mesh. These deformed meshes were then treated as data and were used directly for comparisons with simulation results. By performing parameter sweeps, computing and minimizing $\mathcal {J}$ over a bounded parameter space, we obtained parameter estimates and quantified the behavior of $\mathcal {J}$.

#### LV diastolic filling

Several constitutive laws (see Sect. 2.2.2) were employed to model the passive behavior of the tissue (step “SIMULATION” in Fig. 1), and the simulated diastolic deformation in each case was compared with diastolic data to provide parameter estimates. The LV was modeled as a thick-walled truncated ellipsoid of typical cardiac dimensions. The domain was discretized using a mesh composed of $56$ hexahedral elements, with two elements transmurally, and a quadratic linear interpolation scheme was employed for the displacement and pressure variables, respectively. A generic heterogeneous fiber field was applied, with the fiber angle varying linearly between $60^{\circ }$ and $-60^{\circ }$ from endocardium to epicardium. A zero traction condition was enforced on the epicardial surface, while the base plane was fully fixed. Finally, the endocardial surface of the LV model was passively inflated to a typical end-diastolic pressure of 1.5 kPa, using 50 uniform loading steps.

#### In silico assessment protocol

As we are interested in the passive cardiac parameters, we have generated synthetic tags (step “SYNTHETIC TMRI” in Fig. 1) from a passive inflation simulation of a model left ventricle (LV) (see Sect. 2.1.1) using the various constitutive laws, which will be described in Sect. 2.2.2. Using rasterization, a binary mask of the mesh was created, and tag planes were generated within the image (Sermesant et al. 2003; Duan et al. 2007), resulting in a final 3D tagged image of resolution $1 \times 1 \times 1$ mm. Simulated deformations were then mapped and interpolated within the image, producing a 3D-tagged representation of our passive inflation simulation. For the remaining steps of the parameter estimation study, these synthetic tags served as our data and were treated as real 3D tags.

In order to assess the effect of data noise, Gaussian noise was added to the simulation results, prior to the in silico tagging (step “UNBIASED NOISE” in Fig. 1). The mean value of the added Gaussian noise was zero, and the variance was a percentage (usually 5–20 %) of the maximum deformation of the diastolic simulation.

#### 3D tagged MRI/in silico tagged motion extraction

Myocardial motion was extracted from 3D tagged and in- silico tagged images (step “MOTION TRACKING” in Fig. 1) using the Image Registration Toolkit.^1^ This software uses a nonrigid registration technique proposed by Rueckert et al. (1999); Schnabel et al. (2001) and subsequently extended to tracking of cardiac motion (Chandrashekara et al. 2004; Shi et al. 2012). The registration algorithm which is based on free-form deformations and optimizing the similarity between two subsequent images allows tracking any point within the myocardium throughout the cardiac cycle and provides the deformation field with respect to the beginning of systole. The obtained deformation fields were then applied on an initial mesh and propagated through time, resulting in a set of deformed meshes, which were used as the “observations” within our parameter estimation process.

#### Mechanical simulations and $\mathcal {J}$ characterization

The parameter estimates for the constitutive laws considered were obtained by parameter sweeps (step “PARAMETER SWEEP” in Fig. 1). Specifically, for each law, we performed passive inflation simulations ( see Sect. 2.1.1) for parameter combinations within a neighborhood of the actual parameters. The parameter estimates were then obtained as the set of values that minimized the objective function $\mathcal {J}$ over the parameter space. Within this process, 10 synthetic tagged frames were used as observations. The objective function employed—defined and discussed in Sect. 2.3.2—is discerning and thus able to provide a unique minimum, assuming that the constitutive law is practically identifiable. Note that as the same constitutive law is used for the generated data and simulations, the estimation process does not suffer from model fidelity issues, leading to safe conclusions about practical identifiability.

#### In vivo $\mathcal {J}$ characterization

The study was then extended to an in vivo case of a healthy volunteer, to allow for the assessment of practical identifiability and model fidelity in a real-world setting.

The data used in this study were acquired from a healthy 28-year old male volunteer with a normal heart function. The geometry and cardiac motion were characterized using cine MRI images in retrospective ECG gating (short-axis view, acquired spatial resolution $2 \times 2 \times 8$ mm, temporal resolution $\sim$20 ms), and 3D tagged MRI images in prospective ECG triggering (acquired in 3 breath-holds and reconstructed to spatial resolution of $1 \times 1 \times 1$ mm, temporal resolution $\sim$30 ms). The myocardial motion was extracted from the 3D tagged MRI images using the algorithm described in Sect. 2.1.3.

The cubic Lagrange hexahedral mesh used for the simulations was based on the end-systolic frame of the data (Wang et al. 2009) and was composed of 72 elements, with two elements transmurally. This mesh was created by first constructing a cubic hexahedral end-diastolic mesh (Lamata et al. 2011) from a short-axis CINE MRI stack registered to the 3D tagged MRI images using the IRTK imaging toolkit (see Sect. 2.1.3). The mesh was then uniformly refined into a linear end-diastolic (ED) mesh consisting of 96,768 hexahedral elements. Nodes of the linear ED mesh were then tracked through the cardiac cycle, providing the deformed LV geometry at end systole. This deformed ED mesh was used as a template for the coarser 72 element end-systolic (ES) cubic mesh, which was generated by least-square fitting. Observations—in this case displacements from end systole—were then required to compute the objective function (see Eq. 15). These were extracted from the linear ED mesh by subtracting the displacements observed at the end-systolic frame from the diastolic displacements. These computed displacements at the linear ED mesh nodes were then mapped onto a linear version of the ES mesh by doing a nearest point search (mean point search error 0.8 mm). A large number of elements were used in the linear ED mesh to minimize potential errors due to the mapping of data. The final projected displacements on the linear ES mesh served as observations within our parameter estimation process.

The cubic end-systolic mesh was inflated by prescribing the data-derived cavity volume at each time step, instead of inflating by pressure as used in the in silico tests. This was due to the lack of cavity pressure measurements for this in vivo test. The volume constraint was enforced weakly through a Lagrange multiplier. The base motion was prescribed directly from the observations—13 diastolic frames based on increasing cavity volume—and zero traction was applied on the epicardial surface. The myocardial model was assumed to be incompressible (see Sect. 2.2.1), even though potential compressibility of the extracted myocardial motion was not examined. This is due to the fact that a possible degree of compressibility [2–12 % (Iwasaki et al. 1984)] would be within the level of noise of the tagged MRI data. Running the described simulation with parameter sweeps and comparing with observations, we could then characterize the behavior of $\mathcal {J}$ in a real-world scenario.

### Cardiac mechanics

#### Finite elasticity

The passive diastolic filling of the LV considered in this work was simulated within the finite elasticity framework due to the large deformation of the myocardium during the cardiac cycle (Holzapfel and Ogden 2009).

We consider here a body defined over a reference domain $\varOmega _0$, which deforms under the action of a traction $\varvec{t}$ (such as the endocardial pressure) on a subset $\varGamma ^N$ of the boundary $\partial \varOmega$, where $\varOmega$ is the current configuration. Given a set of parameters $\varvec{\theta }$ related to the employed constitutive law, the mechanics problem can be written as: Find the deformation and hydrostatic pressure pair $x= (\varvec{u}_{\theta }$, $p_{\theta })$ in $\varvec{W}_0 =\varvec{H}_0^1(\varOmega ) \times L^2 (\varOmega )$ such that

1 $$\begin{aligned} \mathcal {F}(\varvec{\theta }; x, y)=0, \quad \forall y \in \varvec{W}_0, \end{aligned}$$

where

$$\begin{aligned} \mathcal {F}(\varvec{\theta }; x, y)&= \int _{\varOmega } (\varvec{\sigma }(\varvec{\theta }; \varvec{u}) + p \varvec{I} ){:}\, \nabla \varvec{v} \; \hbox {d}v \\&\quad -\,\int _{\varGamma ^N} \varvec{t} \cdot \varvec{v} \; da +\int _{\varOmega _0} q (J-1) \; \hbox {d}V . \end{aligned}$$

In the finite elasticity framework considered here, $\varvec{\sigma }$ denotes the deviatoric Cauchy stress tensor. In this setting, $x= (\varvec{u},p )$ and $y= (\varvec{v},q )$ represent the state solutions and test functions, respectively.

The Cauchy stress tensor introduced in Eq. 1 depends on the passive behavior of the material and the constitutive law chosen to describe it. As the myocardium is most typically modeled as a hyperelastic material, the mechanical behavior is expressed using a strain energy function, whose deviatoric component is denoted here by $\varPsi$. The deviatoric component of the Cauchy stress tensor can then be obtained through the expression

2 $$\begin{aligned} \varvec{\sigma }=\frac{2}{J}\varvec{F}\frac{\partial \varPsi }{\partial \varvec{C}}\varvec{F}^{T}. \end{aligned}$$

Here $\varvec{F}$ denotes the deformation gradient defined as

3 $$\begin{aligned} F_{i,j}=\frac{\partial {x_i}}{\partial {X_j}}, \end{aligned}$$

which relates a point in the deformed state $\varvec{x} \in \varOmega$ to its reference configuration $\varvec{X} \in \varOmega _0$ (Bonet and Wood 2008). For volume-preserving materials, incompressibility is enforced using the determinant of $\varvec{F}$ through the constraint $J=\det (\varvec{F})=1$. Additionally, $\varvec{C}$ represents the right Cauchy-Green deformation tensor, defined as $\varvec{C}=\varvec{F}^T\varvec{F}$.

In cardiac mechanics, the finite elasticity problem (1) is commonly solved using the finite element method (FEM) due to the nonlinearities introduced by the constitutive laws and cardiac geometry. The FEM framework is based on discretization of the continuous domain and function spaces (Hadjicharalambous et al. 2014).

#### Constitutive laws

We begin with the Neo-Hookean law, a well-known isotropic hyperelastic law, which has also been employed in cardiac models (Cheng et al. 2005). The strain energy function $\varPsi$ for the Neo-Hookean law is defined as

4 $$\begin{aligned} \varPsi = \frac{\mu }{2}(I_{\hat{C}}-3), \end{aligned}$$

where $\mu$ is the stiffness of the material, $\hat{\varvec{C}}= J^{-\frac{2}{3}} \varvec{C}$ is the isochoric component of $\varvec{C}$ and $I_{\hat{C}}$ is the first invariant of $\hat{\varvec{C}}$ ($I_{\hat{C}}= \hat{\varvec{C}}{:}\, \varvec{I}$). The deviatoric stress can then be expressed as

5 $$\begin{aligned} \varvec{\sigma }= \frac{\mu }{J^{\frac{5}{3}}}\left( \varvec{b}- \frac{I_C}{3}\varvec{I}\right) , \end{aligned}$$

where $\varvec{b}=\varvec{F}\varvec{F}^T$ denotes the left Cauchy-Green deformation tensor.

A more structurally accurate model is then examined, by augmenting the Neo-Hookean law with a fiber-dependent component (Humphrey 2002). This enhanced version which we will refer to as the *Neo-fiber* law is defined with respect to a fiber coordinate system, where the axes are aligned with the fiber $\varvec{{f}_0}$, sheet $\varvec{{s}_0}$ and sheet-normal $\varvec{{n}_0}$ unit vectors. The strain energy function for the Neo-fiber law is defined as

$$\begin{aligned} \varPsi =\frac{1}{2(a+1)}(C_1-C_2)(I_{\hat{C}_f}-1)^{a+1}+\frac{C_2}{2}(I_{\hat{C}}-3), \end{aligned}$$

where $a=1$ or $2$. $C_1$ and $C_2$ are material parameters of the Neo-fiber law, corresponding to the fiber-dependent and isotropic terms, respectively. In this definition, $I_{\hat{C}_f}$ represents the first invariant of $\hat{\varvec{C}}$ in the fiber direction, defined as

6 $$\begin{aligned} I_{\hat{C}_f}= \hat{\varvec{C}}{:}\,\varvec{{f}_0} \otimes \varvec{{f}_0}. \end{aligned}$$

The deviatoric Cauchy stress for the Neo-fiber law is then expressed as

$$\begin{aligned} \varvec{\sigma }&= J^{-\frac{5}{3}}\left[ C_2\varvec{b} +(C_1-C_2)(I_{\hat{C}_f} - 1)^a \varvec{{f}}\otimes \varvec{{f}} \right. \\&\left. \quad - \;\frac{1}{3}\big ( C_2I_C+(C_1-C_2)(I_{\hat{C}_f}-1)^aI_{\hat{C}_f} \big )\varvec{I}\right] \end{aligned}$$

where $\varvec{{f}}= \varvec{F}\varvec{{f}_0}$. To better capture the exponential response of the myocardial tissue, our study is then extended to the structurally based orthotropic law by Holzapfel and Ogden (2009). The deviatoric form of the strain energy function for a 3-dimensional body is defined as

7 $$\begin{aligned} \varPsi&= \frac{a}{2b}\{\exp [b(I_{\hat{C}}-3)]-1\}\nonumber \\&\quad + \; \frac{a_f}{2b_f}\{\exp [b_f(I_{\hat{C}_f}-1)^2] -1\}\nonumber \\&\quad + \; \frac{a_s}{2b_s}\{\exp [b_s(I_{\hat{C}_s}-1)^2] -1\}\nonumber \\&\quad + \; \frac{a_{fs}}{2b_{fs}}[\exp (b_{fs}I_{\hat{C}_{fs}}^2)-1]. \end{aligned}$$

In this definition $I_{\hat{C}_s}= \hat{\varvec{C}}{:}\, \varvec{{s}_0} \otimes \varvec{{s}_0}$ and $I_{\hat{C}_{fs}}= \hat{\varvec{C}}{:}\,\varvec{{f}_0} \otimes \varvec{{s}_0}$ denote invariants associated with the sheet and cross-fiber directions.

In what follows, we use a reduced version of the Holzapfel–Ogden law, where $a_s$ and $a_{fs}$ are set to zero and the exponents $b$ and $b_f$ are kept constant, as we are restricting this study to constitutive laws with a small number of parameters to allow for better identifiability. A similar formulation has also been applied in (Caruel et al. 2014) in 0D and 1D models, demonstrating its ability to fit experimental data and thus reproduce physiological cardiac behavior. The values of the exponents ($b=5$ and $b_f=5$) are chosen so that the model is able to provide a physiological EDPVR (see Sect. 3.3 and Fig. 12), as we note that for several combinations of $b$ and $b_f$ a physiological EDPVR could not be produced for any values of the scaling parameters $\alpha$ and $\alpha _f$. Nevertheless, there is a range of values that would be appropriate for this choice, as we can assume interdependence between the exponents and scaling constants similar to that of the Guccione law (Xi et al. 2011a). The added value of this formulation over the Guccione law is that due to its structure as a sum of individual exponential terms, it can be reduced into a form with more than one uncoupled parameters. For this reduced version, the deviatoric Cauchy stress is derived as follows:

8 $$\begin{aligned} \varvec{\sigma } \,&= \,J^{-\frac{5}{3}}\big [a\exp [b(I_{\hat{C}}-3)]\varvec{b}\nonumber \\&\quad + \; 2a_f(I_{\hat{C}_f}-1)\exp [b_f(I_{\hat{C}_f}-1)^2] \varvec{{f}}\otimes \varvec{{f}}\nonumber \\&\quad - \; \frac{1}{3}\big ( a \exp [b(I_{\hat{C}}-3)]I_C\nonumber \\&\quad + \; 2a_f(I_{\hat{C}_f}-1)\exp [b_f(I_{\hat{C}_f}-1)^2]I_{\hat{C}_f} \big ) \varvec{I} \big ]. \end{aligned}$$

Finally, we examine the well-known transversely isotropic exponential law by Guccione et al. The strain energy function $\varPsi$ is defined with respect to a fiber-oriented Green–Lagrange strain tensor $\varvec{E}_F$

9 $$\begin{aligned} \varvec{E}_F = \varvec{Q}^T \varvec{E} \varvec{Q}= \left( \begin{array}{c@{\quad }c@{\quad }c} E_{ff} &{} E_{fs} &{} E_{fn} \\ E_{sf} &{} E_{ss} &{} E_{sn} \\ E_{nf} &{} E_{ns} &{} E_{nn} \\ \end{array} \right) , \end{aligned}$$

where the rotation tensor $\varvec{Q}$ is defined as $\varvec{Q}=[\varvec{{f}_0}, \varvec{{s}_0}, \varvec{{n}_0}]$. The strain energy function is then defined as

$$\begin{aligned} \varPsi = \frac{1}{2}C(e^Q -1), \quad Q = (\varvec{a} \circ \varvec{E}_F){:}\,\varvec{E}_F, \end{aligned}$$

where $\varvec{a}$ is a matrix of constants describing the degree of anisotropy in each component:

$$\begin{aligned} \varvec{a} = \left( \begin{array}{c@{\quad }c@{\quad }c} b_f &{} b_{fs} &{} b_{fs} \\ b_{fs} &{} b_t &{} b_t \\ b_{fs} &{} b_t &{} b_t \\ \end{array} \right) . \end{aligned}$$

The Cauchy stress tensor can then be expressed as

10 $$\begin{aligned} \varvec{\sigma } = J^{-1}\varvec{F} C e^Q \varvec{Q} ( \varvec{a} \circ \varvec{E}_F ) \varvec{Q}^T\varvec{F}^T. \end{aligned}$$

### Parameter estimation

In patient-specific mechanics simulations, we often wish to tune or parameterize our models based on measurement data (observations). Supposing we have $N$ parameters which govern the material behavior, it is a common approach to try and parameterize based on objective function minimization. For example, we aim to find a set of $N$ parameters $\varvec{\theta }_{\mathrm{min}}$ which satisfies, for an objective function $\mathcal {J}_\theta$,

11 $$\begin{aligned} \mathcal {J}_\theta (\varvec{\theta }_{\mathrm{min}}) < \min _{\varvec{\theta }\in P \backslash \varvec{\theta }_{\mathrm{min}} } \mathcal {J}_\theta (\varvec{\theta }) \end{aligned}$$

where $P \subseteq \mathbb {R}^N$ is a subset of vectors of real numbers which constitute the admissible parameters for the problem. The behavior of the model, the observations (or data) over which the behavior is considered, and the objective function itself all play an important role in the behavior of the minimization problem and uniqueness of the minimizer. This is particularly important in clinical contexts where the obtained set of parameters $\varvec{\theta }_{\mathrm{min}}$ is used to, in some sense, provide an indicator of the health/state of the myocardium. In this section, we examine how these factors—the model, observations and objective—can influence the identifiability of $\varvec{\theta }_{\mathrm{min}}$.

#### Model identifiability

To understand the behavior of the minimization problem, we first aim to better understand the behavior of the model and its parameters. Suppose we consider $N_s$ loading conditions imposed on our model (shown in Eq. 1). In this case, we can write the total model problem using the operator $\mathcal {F}_s$ where we sum each quasi-static equilibrium state defined in Eq. 1,

12 $$\begin{aligned} \mathcal {F}_s(\varvec{\theta }; X, Y)&= \sum _{k=1}^{N_s} \int _{\varOmega _k} \varvec{\sigma } (\varvec{\theta }; \varvec{u}_k) : \nabla \varvec{v} _k \; \varvec{dx} \nonumber \\&\quad \; + \; \int _{\varOmega _k} p_k \nabla \cdot \varvec{v} _k \varvec{dx} \nonumber \\&\quad \; + \; \int _{\varOmega _0} q_k (J(\varvec{u}_k) - 1) \varvec{dX}\nonumber \\&\quad \; - \; \int _{\varGamma _k^N} \text{ t }_k \cdot \varvec{v} _k \varvec{dx} . \end{aligned}$$

In this notation $\{ \text{ t }_1, \ldots \text{ t }_{N_s} \}$ denotes the set of $N_s$ loading conditions (boundary tractions) and

$$\begin{aligned} X&= \{ \varvec{u}_1, \ldots \varvec{u}_{N_s} \} \times \{ p_1, \ldots p_{N_s} \}\\ Y&= \{ \varvec{v} _1, \ldots \varvec{v} _{N_s} \} \times \{ q_1, \ldots q_{N_s} \} \end{aligned}$$

denote the set of state solutions and test functions for each load state $k$. We can also compose the solution spaces setting $X,Y \in \varvec{W}_0^s$, where the space $\varvec{W}_0^s = \left[ \varvec{H}_0^1(\varOmega ) \right] ^{N_s} \times \left[ L^2 (\varOmega ) \right] ^{N_s}$ is an extension of space $\varvec{W}_0$ accounting for the multiple loading states. Additionally, in what follows, $W_{0}^{s}=W_{0}^{d}\times W^{p}$, where spaces $W_{0}^{d}$ and $W^{p}$ correspond to displacement and pressure variables, respectively.

Using this notation, our quasi-static mechanics problem is (given a set of parameters $\varvec{\theta }$): find an $X_\theta \in \varvec{W}_0^s$ such that,

$$\begin{aligned} (P1) \quad \mathcal {F}_s(\varvec{\theta }; X_\theta , Y) = 0, \quad \forall Y \in \varvec{W}_0^s. \end{aligned}$$

Here $$ X_\theta $$ constitutes the state solution composed of displacements and pressures at each of the $$N_s $$ loading conditions. We can further collect all solutions to $$ (P1) $$ and construct a space of solutions $$\varvec{\mathcal {V}}\subset \varvec{W}_0^s$$, i.e.,

$$\begin{aligned} \varvec{\mathcal {V}}= \{ X \in \varvec{W}_0^s | \; \exists \varvec{\theta }\in P \text { s.t. } (X,\varvec{\theta }) \text { satisfy } (P1) \} . \end{aligned}$$

**Fig. 3:**
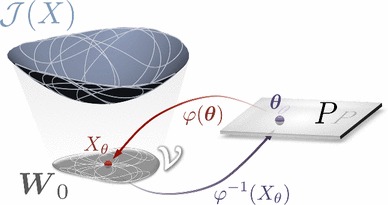
Schematic representation of the objective function $\mathcal {J}$ over the solution space $W_0$ for a given parameter set $\varvec{\theta }$. The bijectivity of mapping $\varphi$ between parameter space ($P$) and space of solutions ($\varvec{\mathcal {V}}$) ensures practical identifiability

From the definition of $\varvec{\mathcal {V}}$, we observe that we can identify pairings between a subset of $P_{\varvec{\mathcal {V}}} \subseteq P$ and the space of state solutions $\varvec{\mathcal {V}}$. These pairings, in general, have no well-defined properties. Indeed, $\varvec{\mathcal {V}}$ and $P_{\varvec{\mathcal {V}}}$ may be empty. Here we suppose that $(P1)$ induces a surjective mapping on the parameter space $P$ to the space of state solutions $\varvec{\mathcal {V}}$ (see Fig. 3), i.e.,

$$\begin{aligned} \varphi {:}\,P \mapsto \varvec{\mathcal {V}}, \qquad \varphi (\varvec{\theta }) = X_\theta , \quad (\varvec{\theta },X_\theta ) \in P \times \varvec{\mathcal {V}}\end{aligned}$$

This assumption is equivalent to assuming that there exists a unique $X_\theta \in \varvec{\mathcal {V}}$ for every $\varvec{\theta }\in P$. If $\varphi {:}\,P \mapsto \varvec{\mathcal {V}}$, it implies that for any $\varvec{\theta }\in P$ there is an $X_\theta \in \varvec{\mathcal {V}}$. In other words, it implies there exists a $(X_\theta , \varvec{\theta })$ satisfying (P1). Moreover, non-uniqueness of solutions would imply that for some $\varvec{\theta }\in P$, the best we could do to write the mapping $\varphi$ is to write

$$\begin{aligned} \varphi (\varvec{\theta }) = \{ X_\theta ^1, X_\theta ^2, \ldots \} \subseteq \varvec{\mathcal {V}}. \end{aligned}$$

This possibility is precluded, however, by the surjective assumption (implying uniqueness). Though it is not proven for general cardiac mechanics boundary value problems, it is often assumed in these applications that, given the admissible set of loading states and parameters, the solution $X_\theta$ to $(P1)$ exists and is unique.

However, this condition is insufficient to guarantee unique parameter identifiability in Eq. 11. A stronger condition, which we show can ensure unique parameter identifiability, occurs when the mapping $\varphi$ is in fact bijective, i.e., there exists a $\varphi ^{-1}$ where

$$\begin{aligned} \varphi ^{-1}{:}\,\varvec{\mathcal {V}}\mapsto P, \qquad \varphi ^{-1} (X_\theta ) = \varvec{\theta }. \end{aligned}$$

In the case where $\varphi$ is bijective, we can ensure that the transition from state to parameters is well defined.

Two important determinants of the properties of $\varphi$ stem from the behavior of the constitutive law itself and the set of loading states. The parameter dependence of the constitutive law—whether it be linear or nonlinear—can significantly influence a model’s ability to uniquely identify parameters. It may also influence the range of loading states (and, as a result, deformations) which must occur to elucidate parameter dependencies. The final set of $N_s$ loading states $\{\text{ t }_1, \ldots \text{ t }_{N_s}\}$ then defines this scope of deformations. For example, in the case of pure tension of the orthotropic Costa law, while fiber, sheet and sheet-normal parameters influence the end solution, the non-diagonal strain terms have no influence.

These considerations lead to a basic property required of a model referred to as *structural identifiability* (Raue et al. 2009, 2011). A model is said to be structurally identifiable if there exists an arbitrarily large set of $N_T$ loading states $\{ \text{ t }_1, \ldots \text{ t }_{N_T} \}$ such that the mapping $\varphi {:}\,P \mapsto \varvec{\mathcal {V}}$ is bijective. As we show below (see Theorem 2 and Appendix 1), this property is easily proven for constitutive laws with linear parameter dependence, but becomes more complex when this dependence is nonlinear.

However, in most in vivo scenarios, model parameterization is limited to a given set of loading states which cannot be arbitrarily selected, leading to the concept of *practical identifiability*. A model is said to be practically identifiable if, for a given set of $N_s$ loading states $\{ \text{ t }_1, \ldots \text{ t }_{N_s} \}$, the mapping $\varphi {:}\,P \mapsto \varvec{\mathcal {V}}$ is bijective. The key difference here is that limited observations comprise a set of loading states that ensure identifiability of all parameters, $\varvec{\theta }$. In our case, these represent the in vivo states observed through the cardiac cycle which must be sufficient to yield practical identifiability of all parameters of the law.

For general nonlinear cardiac models, practical identifiability of passive parameters is difficult to prove a priori as it fundamentally depends on the structural identifiability of the model and the set of loading states and observations provided by the data. However, these considerations dictate the choice of model best suited for a given set of material deformations.

In general, bijectivity can be ensured by a coercivity assumption, i.e.,

##### **Theorem 1**

Suppose $\varphi {:}\, P \mapsto \varvec{\mathcal {V}}$ is a surjection (i.e., the solution to (P1) exists and is unique). Then if for any $X \in \varvec{\mathcal {V}}$ a pair $\varvec{\theta }_1,\varvec{\theta }_2 \in P$,

$$\begin{aligned}&\alpha \Vert \varvec{\theta }_1 - \varvec{\theta }_2 \Vert _P \le \\&\qquad \qquad \qquad \sup _{Y \in \varvec{W}_{0,Div}^u} \frac{| \mathcal {F}_s(\varvec{\theta }_1; X, Y) - \mathcal {F}_s(\varvec{\theta }_2; X, Y) |}{\Vert Y\Vert _{\varvec{W}_{0}^u}} \end{aligned}$$

then $\varphi$ is bijective.

##### *Proof*

The proof follows from contradiction. Suppose that $\varvec{\theta }_1,\varvec{\theta }_2 \in P$ both happen to satisfy (P1) when paired with the solution states $X$. Then by $(P1)$,

$$\begin{aligned} 0&= \mathcal {F}_s(\varvec{\theta }_1; X, Y) - \mathcal {F}_s(\varvec{\theta }_2; X, Y), \quad \forall Y \in \varvec{W}_{0}^u. \end{aligned}$$

Dividing both sides by $\Vert Y \Vert _{\varvec{W}_{0}^u}$ and taking the absolute value and supremum, the coercivity assumption ensures,

$$\begin{aligned} 0 \ge \Vert \varvec{\theta }_1 - \varvec{\theta }_2 \Vert _P \end{aligned}$$

or that the parameters $\varvec{\theta }_1$ and $\varvec{\theta }_2$ are, in fact, the same. Hence, any solution $X$ has a single pair $\varvec{\theta }\in P$.$\square$

A much simpler case occurs when the model depends linearly on $N_p$ parameters, the properties of $\varphi$ are easier to decipher. In this case, the model may be written as:

13 $$\begin{aligned} \varvec{\sigma }( \varvec{\theta }; \varvec{u}) = \sum _{n=1}^{N_p} \theta _n \varvec{\sigma }_n(\varvec{u}), \end{aligned}$$

where $$ \varvec{\sigma }_n $$ is the stress tensor (which may nonlinearly depend on $$ \varvec{u}$$) scaled by the $$ n $$th parameter. In this case, the bijectivity of $$\varphi $$ can be ensured by guaranteeing that it is possible to construct $$ N $$-constraints by using different $$Y$$’s in $$(P1) $$. Due to the linear parameter dependence, the constraints may then be written as a matrix system, where the invertibility of the matrix ensures $$\varphi $$ is bijective (see Theorem 2).

##### **Theorem 2**

Suppose $\varphi {:}\,P \mapsto \varvec{\mathcal {V}}$ is a surjection (i.e., the solution to (P1) exists and is unique). If there exists a set of functions $\{ Y_1, \ldots Y_N \}$, $Y_i \in \varvec{W}_{0,Div}^u$ with

$$\begin{aligned} Y_i = \{ \varvec{v} _1^i, \ldots \varvec{v} _{N_s}^i \} \end{aligned}$$

such that the matrix $\varvec{A}$ with entries

$$\begin{aligned} A_{ij} = \sum _{k=1}^{N_s} \int _{\varOmega _k} \varvec{\sigma }_j (\varvec{u}_k){:}\,\nabla \varvec{v} _k^i \; \varvec{dx} \end{aligned}$$

is invertible, then $\varphi$ is bijective.

##### *Proof*

The proof may be shown, again, by contradiction. Suppose there are two sets of nonidentical parameters $$ \varvec{\theta }, \varvec{\psi } \in P $$ which result in the same state $$ X$$. Then, by $$ (P1) $$,

14 $$\begin{aligned} 0&= \mathcal {F}_s(\varvec{\theta }; X, Y) - \mathcal {F}_s(\varvec{\psi }; X, Y) \nonumber \\&= \sum _{n=1}^{N_p} (\theta _n - \psi _n) \sum _{k=1}^{N_s} \int _{\varOmega } \varvec{\sigma }_n (\varvec{u}_k){:}\,\nabla \varvec{v} _k \; \hbox {d}v \end{aligned}$$

for any $Y \in \varvec{W}_{0,Div}^u \times ( \varvec{W}^p \cap \varvec{0})$. Hence, choosing $\{ Y_1, \ldots Y_{N_p} \}$, Eq. 14 may be rewritten as

$$\begin{aligned} \varvec{A} (\varvec{\theta }- \varvec{\psi }) = 0 \quad \Rightarrow \quad \varvec{\theta }= \varvec{\psi } \end{aligned}$$

due to the invertibility of $\varvec{A}$.$\square$

Theorem 2 depends on a sufficient number of deformation states such that $\varvec{A}$ gains linearly independent rows. We then rely on *any* test functions in $\varvec{W}_{0,Div}^u$ to further accentuate differences in material response, providing a flexible source from which to select constraints. Using this theorem, we can prove the structural identifiability for the Neo-Hookean, Neo-fiber and reduced Holzapfel–Ogden law which have a linear dependence on their parameters (see Appendix 1).

#### Objective function-based minimization

In data-based parameter estimation procedures, we often rely on some objective function to guide the choice of parameters. Since the parameters are not observed in most cases, we rely instead on comparing states with observations. In these cases, it is necessary that the objective function $\mathcal {J}{:}\,\varvec{\mathcal {V}}\rightarrow \mathbb {R}$ obtains a unique minimum (a *discerning objective*).

Using $3D$ tagged data where the states are usually displacements, the natural choice of objective function is to use the $\varvec{L}^2$ norm over all states, i.e.,

15 $$\begin{aligned} \mathcal {J}( X ) = \frac{ ||| X - X_d ||| }{ ||| X_d |||} \end{aligned}$$

where $X_d = \{ \varvec{u}_1, \ldots \varvec{u}_{N_s} \}$ are observations on the displacements in the myocardium and we divide through by the overall scale of displacements so that $\mathcal {J}$ gives a percentage error. In this case, $||| \cdot |||$ is a norm on $\varvec{W}_0^{d}$ defined as,

$$\begin{aligned} ||| X ||| = ((X,X))^{1/2} , \quad ((X, Y )) = \sum _{k=1}^{N_s} ( \varvec{u}_k, \varvec{v} _k ) \end{aligned}$$

where $(\cdot , \cdot )$ is the $\varvec{L}^2-$inner product on the reference domain $\varOmega _0$. We then look to minimize the objective, finding $X_{\mathrm{min}} \in \varvec{\mathcal {V}}$ where

16 $$\begin{aligned} \mathcal {J}(X_{\mathrm{min}}) < \min _{X \in \varvec{\mathcal {V}}\backslash X_{\mathrm{min}} } \mathcal {J}(X). \end{aligned}$$

As $||| \cdot |||$ acts as a norm on displacements in $\varvec{\mathcal {V}}$ (and a semi-norm on the entire space), if the observed displacements in the state $X_d$ constitute a set of displacements $\widetilde{X} \in \varvec{\mathcal {V}}$, then $\mathcal {J}$ is automatically a discerning objective as the norm has a unique zero by definition (and is strictly nonnegative).

This is, however, unlikely to occur in a real context due to two dominant factors: (1) data noise and resolution, (2) model fidelity. The introduction of noise, or degradation in data due to image resolution, introduces offsets which make the likelihood of $X_d$ being a member of $\varvec{\mathcal {V}}$ minimal. In addition, the fidelity of the model can strongly influence whether or not the model can capture the behavior observed in the data, making it possible that one or more than one minima exist.

Supposing that the model is a good representation of the tissue in vivo, we can then write $X_d = \widetilde{X} + \varepsilon$. In this case, if we assume that $\varepsilon$ is, in fact, some random unbiased noise which satisfies

17 $$\begin{aligned} (( X, \varepsilon )) \approx 0, \quad \forall X \in \varvec{\mathcal {V}}, \end{aligned}$$

then we observe that the noise does not bias our minima, but instead introduces a constant offset in $\mathcal {J}$, i.e.,

18 $$\begin{aligned} \mathcal {J}(X) = \frac{ ||| X - X_d ||| }{ ||| X_d ||| } = \frac{ \left( ||| X - \widetilde{X} |||^2 + ||| \varepsilon |||^2 \right) ^{1/2} }{ ||| X_d ||| } . \end{aligned}$$

The assumed relation in Eq. 17 is a reasonable assumption when the noise fluctuations occur over a small spatial scale compared with the change of the state solutions $X$ near the minima.

The offset in Eq. 18 does not influence the minimization on $\varvec{\mathcal {V}}$ and, as a result, $\mathcal {J}$ remains a discerning objective. Obtaining a unique minimum in $\varvec{\mathcal {V}}$ is essential as even if a model is practically identifiable based on loading constraints, multiple minima for $\mathcal {J}$ guarantee multiple minima in parameter space. However, with a discerning objective, we then rely on the bijectivity (or practical identifiability) of $\varphi$ so that the objective formed through composition,

19 $$\begin{aligned} \mathcal {J}_\theta ( \varvec{\theta }) = \mathcal {J}\circ \varphi (\varvec{\theta }) \end{aligned}$$

also obtains a unique minimum

20 $$\begin{aligned} \mathcal {J}_\theta ( \varvec{\theta }_{\mathrm{min}}) < \min _{\varvec{\theta }\in P \backslash \varvec{\theta }_{\mathrm{min}} } \mathcal {J}_{\theta } ( \varvec{\theta }). \end{aligned}$$

In practice, we can see that a discerning objective and a set of load states giving practical identifiability are sufficient conditions to ensure that the set of parameters $\varvec{\theta }_{\mathrm{min}}$ are uniquely identifiable.

#### Parameter coupling

Characterizing the behavior of the objective function $\mathcal {J}$ over the parameter space is critical for the performance of the parameter estimation process. The behavior of $\mathcal {J}$ around the minimum value (a distinct localized minimum instead of a wide valley) indicates a discerning objective function, which would allow data assimilation methods to retrieve the parameter estimate. Further, the landscape of $\mathcal {J}$ over the parameter space provides important information regarding the practical identifiability of the constitutive law, revealing the presence of a unique or multiple minima or possible inter-parameter coupling.

Coupling can also be deduced by the Hessian matrix of the objective function at the obtained minimum. Using the Taylor expansion of $\mathcal {J}$ around the obtained minimum $\varvec{\theta }_{\mathrm{min}}$,

$$\begin{aligned} \mathcal {J}( \varvec{\theta }_{\mathrm{min}}+\varvec{\epsilon })&= \mathcal {J}( \varvec{\theta }_{\mathrm{min}}) + \nabla _{\varvec{\theta }} \mathcal {J}( \varvec{\theta }_{\mathrm{min}})^T \varvec{\epsilon } \\&+\,\frac{1}{2}\varvec{\epsilon }^T \nabla _{\varvec{\theta }}\big ( \nabla _{\varvec{\theta }}\mathcal {J}( \varvec{\theta }_{\mathrm{min}}) \big ) \varvec{\epsilon } + \mathcal {O}(||\varvec{\epsilon }||^3). \end{aligned}$$

Due to the gradient being zero at the minimum $\varvec{\theta }_{\mathrm{min}}$,

21 $$\begin{aligned} \mathcal {J}( \varvec{\theta }_{\mathrm{min}}+\varvec{\epsilon })= \mathcal {J}( \varvec{\theta }_{\mathrm{min}}) + \frac{1}{2}\varvec{\epsilon }^T \varvec{H} \varvec{\epsilon } + \mathcal {O}(||\varvec{\epsilon }||^3) \end{aligned}$$

where $\varvec{H}$ denotes the Hessian matrix. As can be deduced by this expression, the Hessian matrix can provide important information as it allows one to relate growth in $\mathcal {J}$ locally to local perturbations in the parameters. Further, to allow comparison between laws with varying parameters’ scale, we use a scaled Hessian $\varvec{\tilde{H}}$ defined as

22 $$\begin{aligned} \tilde{H}_{ij}= H_{ij}\theta _i \theta _j, \end{aligned}$$

where $\theta _i$ and $\theta _j$ correspond to the $i$-th and $j$-th components of $\varvec{\theta }_{\mathrm{min}}$. Then using $\varvec{\epsilon }= \varvec{\tilde{\epsilon }} \circ \varvec{\theta }_{\mathrm{min}}$, Eq. 21 can be expressed as

$$\begin{aligned} \mathcal {J}( \varvec{\theta }_{\mathrm{min}}+\varvec{\epsilon })= \mathcal {J}( \varvec{\theta }_{\mathrm{min}}) + \frac{1}{2}\varvec{\tilde{\epsilon }}^T \varvec{\tilde{H}} \varvec{\tilde{\epsilon }}+ \mathcal {O}(||\varvec{\epsilon }||^3), \end{aligned}$$

where now we are dealing with parameter percentages, which enables comparison between the different laws. The scaled Hessian $\varvec{\tilde{H}}$ can then characterize the sensitivity of $\mathcal {J}$ to the parameters and demonstrate possible inter-parameter coupling. Specifically, the minimum diagonal value of $\varvec{\tilde{H}}$ indicates that $\mathcal {J}$ is least sensitive to the corresponding parameter as a large error in the parameter can result in an insignificant change in $\mathcal {J}$. Similarly, the minimum eigenvalue of $\varvec{\tilde{H}}$ indicates the parameter combination that $\mathcal {J}$ is least sensitive to. Accordingly, the ratio of the minimum diagonal value of $\varvec{\tilde{H}}$ over the minimum eigenvalue $\lambda (\varvec{\tilde{H}})$

23 $$\begin{aligned} R =\frac{\min \{\hbox {diag}(\varvec{\tilde{H}})\}}{\min \{\lambda (\varvec{\tilde{H}})\}} \end{aligned}$$

demonstrates the degree of coupling between parameters. Specifically, large values indicate that there is a parameter combination whose possible error will cause a smaller change in $\mathcal {J}$ than error in each parameter separately, suggesting inter-parameter coupling. Similarly, coupling ratios close to $1$ suggest that there is no significant coupling between parameters.

## Results and discussion

### Comparison of practical identifiability using 3D tags

For each of the considered constitutive laws, the behavior of $\mathcal {J}$ over the parameter space is examined and the error between actual and estimated parameters is quantified. For each law we select a ground truth set of parameters which gives physiologically reasonable end-diastolic volume and pressure and generate synthetic 3D tags from an LV inflation simulation. The extracted myocardial motion applied on meshes is then used as our data and compared with simulations with varying parameter combinations to provide the landscape of the objective function and assess the error in the parameter estimates. Through this process we are able to characterize the practical identifiability of each law and assess its potential use in patient-specific applications.

All tests under consideration were implemented in **CHeart**—a multi-physics software tool based on (Nordsletten 2009; Nordsletten et al. 2010) and expanded by the **CHeart** team at KCL. The problems were solved on a Dell OPTIPLEX 990, quad-core (Intel$^{\circledR }$ Core$^\mathrm{TM}$ i7-2600 CPU @ 3.40 GHz), on a quad-core (Intel$^{\circledR }$ 4th Generation Core$^\mathrm{TM}$ i7-4790 CPU @ 3.60 GHz) and on an 2.1 GHz AMD Opteron$^\mathrm{TM}$ Interlagos 32 processor.

#### $\mathcal {J}$ characterization of the Neo-Hookean law

We begin by investigating the practical identifiability of the Neo-Hookean law, by examining the behavior of $\mathcal {J}$ over a range of stiffness values. Due to the simple structure of the law and its linear parameter dependence, we expect that given some deformation, we should be able to get good identifiability characteristics. Specifically, we expect $\mathcal {J}$ to have a unique and distinct minimum, and we anticipate that the incorporation of unbiased noise should not affect the behavior of $\mathcal {J}$ and the estimated parameter, but only cause a shift toward larger $\mathcal {J}$ values.

**Fig. 4:**
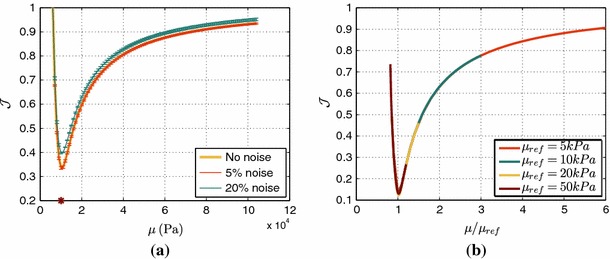
**a** $\mathcal {J}$ over Neo-Hookean stiffness $\mu$, for different data noise levels. The original stiffness value ($\mu =10$ kPa) is marked with a *red asterisk*. The minimum was obtained among $100$ simulations with different stiffness values. **b**: $\mathcal {J}$ over scaled $\mu$ for four different original stiffness values. 100 simulations were performed for each parameter sweep, with an average computational time of 20.518 s

Indeed, as illustrated in Fig. 4a, the objective function $\mathcal {J}$ has a unique and distinct minimum at the initial stiffness value ($\mu = 10$ kPa). Further, the actual stiffness value is retrieved even in the case of noisy data (5 and 20 % Gaussian noise), and the overall behavior of $\mathcal {J}$ remains the same, with just a small shift toward bigger values. These results suggest that using 3D tags we can uniquely and accurately estimate the stiffness value.

The Neo-Hookean in silico test was extended to study the influence of the actual parameter value on the estimation process. Using the same inflating pressure, increased stiffness would result in smaller deformation that might be insufficient to allow for parameter estimation. We therefore performed four passive inflation simulations, where in each case the inflating pressure was adjusted to provide the same end-diastolic volume. We note that as can be observed in Eq. 1 (where the inflating pressure is introduced through traction as the product of pressure and deformed surface normal vector), due to the linear dependence of the law on the parameter the inflating pressure required to produce the same deformation was simply scaled by the ratio between the stiffness values. Figure 4b presents the behavior of the objective function $\mathcal {J}$ over scaled stiffness ($\mu$ over the initial stiffness for each case), showing consistent behavior for any initial stiffness value. This fact confirms practical identifiability of the Neo-Hookean law using 3D tags for any initial stiffness.

#### $\mathcal {J}$ characterization of the Neo-fiber law

**Fig. 5:**
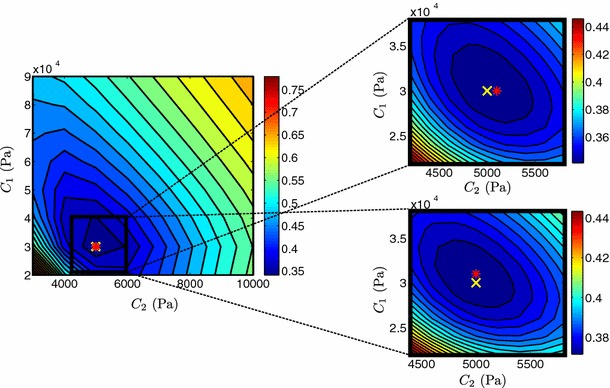
Landscape of objective function $\mathcal {J}$ of Neo-fiber ($a=1$) over parameter space. The actual parameters ($C_1= 30$ kPa, $C_2= 5$ kPa) used in the simulation are shown by a *yellow cross* and estimated parameters by a *red star*. Figures on the *right* show a “zoom-in” near the estimated values, where a denser grid of parameters was used. 20 % Gaussian noise was added in the data, in the *bottom right figure*. 10 $\times$ 10 and 11 $\times$ 11 simulations were performed for the initial and refined parameter sweeps, respectively, with an average computational time of 51.120 s

**Fig. 6:**
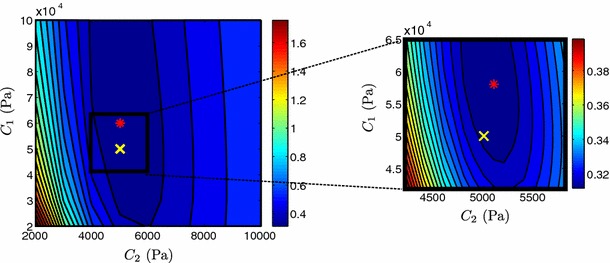
Landscape of objective function $\mathcal {J}$ of Neo-fiber ($a=2$) over parameter space. The actual parameters ($C_1= 50$ kPa, $C_2= 5$ kPa) used in the simulation are shown by a *yellow cross* and estimated parameters by a *red star*. Figure on the *right* shows a “zoom-in” near the estimated values, where a denser grid of parameters was used. 10 $\times$ 10 and 11 $\times$ 14 simulations were performed for the initial and refined parameter sweeps, respectively, with an average computational time of 60.546 s

Figures 5 and 6 illustrate the behavior of the objective function $\mathcal {J}$ over the parameter space of the Neo-fiber law, for $a=1$ and $a=2$, respectively. As can be deduced from Fig. 5, the Neo-fiber law ($a=1$) maintains the practical identifiability of the Neo-Hookean law (distinct minimum) and provides accurate parameter estimates. Even in the case of noisy data, the landscape of $\mathcal {J}$ remains similar and maintains a clear distinct minimum, with a small error (3.3 %) in the fiber parameter.

When the exponent, $a$, is increased to $2$ (see Fig. 6) however, the practical identifiability of the Neo-fiber law is compromised (valley) and the error between actual and estimated parameters increases significantly (2 and 16 % for the isotropic and fiber parameters, respectively). Note that as no noise is added in this case, this error is created during the tagging process due to the combination of limited resolution of the tags and higher nonlinearity of the law.

**Fig. 7:**
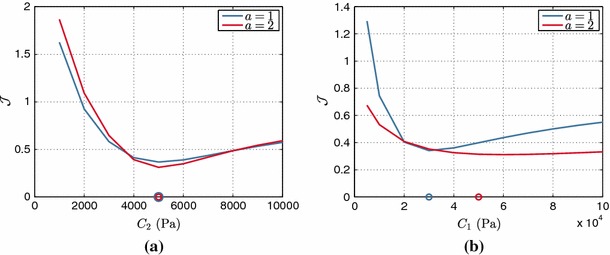
Neo-fiber $\mathcal {J}$ over **a** the isotropic parameter $C_2$ and **b** fiber parameter $C_1$, for $a=1$ and $a=2$. The actual parameters used are marked by the blue and red circles, for $\alpha = 1$ and $\alpha = 2$, respectively

Further, Fig. 7 examines the behavior of $\mathcal {J}$ over a range of values for each parameter separately. The steeper slope of $\mathcal {J}$ around the minimum value in the case of parameter $C_2$ suggests that the isotropic parameter has better identifiability than the fiber parameter. This issue is even more prominent in the $a=2$ case due to the increased nonlinearity in the fiber dependence. Based on Theorem 2, the Neo-fiber law is structurally identifiable due to its linear parameter dependence, suggesting that the deterioration of the identifiability of the fiber parameter is due to insufficient deformation in the data. In fact, when the end-diastolic pressure is increased to $3$ kPa instead of $1.5$ kPa, the error in the fiber parameter decreases from 16 to 12 %.

#### $\mathcal {J}$ characterization of the reduced Holzapfel–Ogden law

**Fig. 8:**
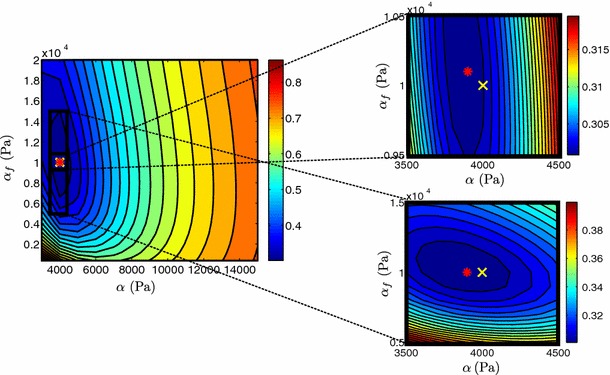
Landscape of objective function $\mathcal {J}$ of reduced Holzapfel–Ogden law over parameter space. The actual parameters ($\alpha = 4$ kPa, $\alpha _f= 10$ kPa) used in the simulation are shown by a *yellow cross* and estimated parameters by a *red star*. Figures on the *right* show “zoom-in” near the estimated values, where a denser grid of parameters was used. 12 $\times$ 14 and 11 $\times$ 12 simulations were performed for the initial and refined parameter sweeps, respectively, with an average computational time of 56.705 s

**Fig. 9:**
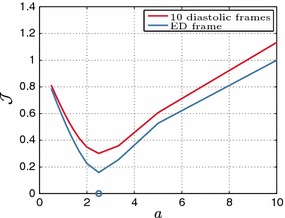
$\mathcal {J}$ over parameter ratio $\alpha _f/\alpha$ for the reduced Holzapfel–Ogden law, when 10 or only the final diastolic frames are considered within $\mathcal {J}$. The actual ratio is marked by a *circle*

The practical identifiability of the reduced Holzapfel–Ogden model (see definition in Eq. 8) was tested setting the values of the exponents ($b=5$ and $b_f=5$) to provide a physiological EDPVR (see Sect. 3.3 and Fig. 12). Figure 8 presents the landscape of the objective function $\mathcal {J}$ over the parameter space, indicating a clear and unique minimum for the objective function. Even though the fiber parameter $\alpha _f$ presents deteriorated identifiability characteristics compared with the isotropic parameter $\alpha$ (as also observed in the Neo-fiber case), we are still able to retrieve the parameter values with small relative errors (2.5 and 1 % for the isotropic and fiber parameters, respectively). Further, Fig. 9 which illustrates $\mathcal {J}$ over the parameter ratio $\alpha _f/\alpha$ for varying $\alpha$, indicates the presence of a unique distinct minimum at the actual parameter. In this case, the behavior of $\mathcal {J}$ is also examined when only the end-diastolic frame is taken into account, illustrating that identifiability is preserved even when only one diastolic frame is used.

#### $\mathcal {J}$ characterization of the Guccione law

The practical identifiability of the transversely isotropic Guccione law was tested by choosing the parameters to fit an empirical end-diastolic pressure–volume relation (EDPVR), proposed by Klotz et al. (Klotz et al. 2006) (see Fig. 12). In order to assess the effect of noise in the data, 5 % Gaussian error was added in the simulation results, prior to in silico tagging.

Table 1 presents the 5 parameter combinations with smallest $\mathcal {J}$ values, with and without 5 % Gaussian noise in the data. These combinations vary significantly, suggesting the presence of multiple minima. Indeed, the presence of 5 % noise in the data results in a different estimate of parameters compared with the non-noisy data. Note that this estimate which has a larger difference from the actual simulation parameters, compensates for the increase in $C$ with a decrease in $b_f$, suggesting an inter-parameter dependence.

**Table 1:** The table presents the actual (in bold) and estimated parameters (in italic) for the Guccione law

Actual	$C$	$b_f$	$b_t$	$b_{fs}$	$\mathcal {J}$
	$\varvec{180}$	$\varvec{27.75}$	$\varvec{5.37}$	$\varvec{2.445}$	
No Gaussian error					
	*150*	*35*	*6*	*3*	* 0*.*0049312 *
	250	25	4	3	0.0055157
	250	25	4	5	0.0057497
	200	35	4	3	0.0061345
	300	25	4	2	0.0062534
5 % Gaussian error					
	*250*	*25*	*4*	* 3*	*0*.*0087839*
	150	35	6	3	0.0089282
	250	25	4	5	0.0091885
	200	35	4	3	0.0097995
	300	25	4	2	0.010056

Further, Guccione parameter combinations with 5 smallest error values are presented, with and without 5 % error in the data. Parameter sweeps were performed over 6 different values for each of the four parameters, resulting to 1,296 parameter combinations

**Fig. 10:**
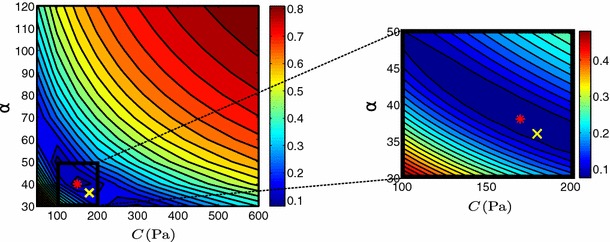
$\mathcal {J}$ over Guccione parameters $C$ and $\alpha$. The actual parameters ($\alpha =38$, $C=180Pa$) used in the simulation are shown by a *yellow cross* and estimated parameters by a *red star*. Figure on the *right* shows a “zoom-in” near the estimated values, where a denser grid of parameters was used. 14 $\times$ 14 and 11 $\times$ 11 simulations were performed for the initial and refined parameter sweeps, respectively, with an average computational time of 66.158 s

In order to further examine this issue, we reformulate the Guccione law as suggested by Xi et al. (2011a):

$$\begin{aligned} b_f=\alpha r_1, \ b_t=\alpha r_2, \ b_{fs}=\alpha r_3, \quad r_1+ r_2+r_3=1, \end{aligned}$$

where parameter $\alpha$ denotes the sum of $b_f$, $b_t$, $b_{fs}$. We performed parameter sweeps over parameters $C$ and $\alpha$ while keeping the ratios between $b_f$, $b_t$, $b_{fs}$ and $\alpha$ constant ($r_1= 0.5, \ r_2=0.3,\ r_3=0.2$). Figure 10 illustrates the behavior of the objective function $\mathcal {J}$ over a range of the parameters $C$ and $\alpha$. The exponential shape of the blue valley representing model parameters resulting in small $\mathcal {J}$ values, verifies coupling between $C$ and $\alpha$ as previously reported by Xi et al. Xi et al. (2011a, 2013). The presence of inter-parameter dependence is also demonstrated in Table 2 which shows a significantly larger coupling ratio R (see Sect. 2.3.3) for the Guccione law. Coupling may significantly deteriorate the parameter estimation process, as any noise in the data is likely to result in a large error in the estimated parameters (in this case 5.6 % for both $\alpha$ and $C$). The coupling in the Guccione law therefore suggests that we cannot guarantee unique and reliable parameter estimates using 3D tags.

**Table 2:** Gradients at the obtained minima, for the considered constitutive laws

	Neo-Hookean	Neo-fiber $a=1$	Neo-fiber $a=2$	r. Holzapfel–Ogden	Guccione
Gradient	$-$1.2516e$-$05	$-$2.6958e$-$07	4.8918e$-$08	$-$7.3606e$-$06	$-$2.7133e$-$04
		$-$2.4444e$-$06	7.7456e$-$06	$-$4.4053e$-$07	$-$2.2966e$-$03
*R*		1.3962	1.1848	1.2586	16.596
*R* (20 % noise )		1.4006	1.2019	1.3001	20.883

First row corresponds to the gradient with respect to the first parameter for each law. Coupling ratios *R* are also presented, for data with and without 20 % unbiased noise

#### Effect of noise in the 3D tagged data

The practical identifiability of the constitutive laws considered may be significantly compromised by the presence of noise in the data. In order to assess this effect, we have considered noisy data, where unbiased Gaussian noise was added in the simulation results prior to in silico tagging. Due to the limited resolution of the data, the addition of noise is expected to deteriorate the behavior of $\mathcal {J}$, especially for parameters with very low sensitivity. The presence of noise resulted in increased $\mathcal {J}$ values (see Figs. 4a, 5) and larger errors in the parameter estimates as indicated in Tables 1 and 3. However, the landscape of $\mathcal {J}$ was not significantly altered due to the uniform noise used, as indicated by the representative case of Neo-fiber $a=1$ in Fig. 5. Nonetheless, unbiased noise caused a minor change to the topology of the objective function in parameter space as can be deduced by the increase in the coupling ratio in Table 2.

**Table 3:** Percentage error (PE) between actual and estimated parameters for each law

	Neo-Hookean (%)	Neo-fiber $a=1$ (%)	Neo-fiber $a=2$ (%)	r. Holzapfel–Ogden (%)	Guccione (%)
PE	0 $\pm$ 10	$0 \ \pm 3.33$	$16 \ \pm 2$	$2.5 \ \pm 2.5$	$5.56 \ \pm 5.56$
		$2 \ \pm 2$	$2 \ \pm 2$	$2 \ \pm 1$	$5.56 \ \pm 5.56$
PE (20 % noise)	$0 \pm 10$	$3.33 \pm 3.33$	$16 \ \pm 2$	$5 \ \pm 2.5$	$5.56 \ \pm 5.56$
		$0 \ \pm 2$	$0 \ \pm 2$	$5 \ \pm 1$	$0 \ \pm 5.56$

The interval used in each parameter sweep is used as the uncertainty in each case. First row corresponds to the error of the first parameter for each law

### Comparison of models’ fidelity

Keeping in mind that the choice of an appropriate constitutive law should balance between parameter identifiability and model fidelity, the constitutive laws described in Sect. 2.2.2 are tested with respect to their ability to represent physiological cardiac deformation. Further, the behavior of the objective function for any constitutive law is also influenced by model fidelity (see Sect. 2.3.2), as a model which cannot provide a good approximation to data can lead to unreliable parameters. We note that even though model fidelity does not affect the in silico tests in Sect. 3.1, where the same constitutive law is used for generated data and simulations, it is an issue for in vivo cases where the model should represent cardiac deformation.

For the purposes of this comparison, Guccione deformations were considered as the ground truth for physiological cardiac deformation. In order to cover a range of possible deformation modes during the cardiac cycle, 18 widely varying parameter combinations were used. For each of these combinations, a parameter sweep was performed for each law to provide the parameter that minimizes the difference between simulations and the ground truth cardiac deformation. The comparison was performed on simulations with the same end-diastolic volume through volume-prescribed loading to reduce the parameter space by 1.

**Fig. 11:**
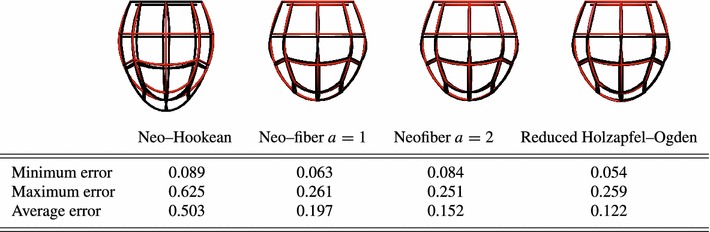
Neo-Hookean, Neo-fiber ($a=1$ and $a=2$) and reduced Holzapfel–Ogden laws are compared in terms of their ability to approximate physiological cardiac deformation. 18 Guccione parameter combinations are used as ground truth. Shown in *black* are the ground truth deformations and in red the best fit over the specified models’ parameter space. Plots show the worst match for all 18 Guccione parameter combinations. The table presents minimum, maximum and average $\mathcal {J}$ values over the 18 Guccione combinations for the various laws

Figure 11 compares the minimum, maximum and average errors between the various laws considered and ground truth deformation. The Neo-Hookean law exhibits a significant error compared with ground truth deformation. This is mainly due to the inability of the Neo-Hookean law to produce adequate elongation and twist, which are important characteristics in cardiac deformation. On the other hand, the added fiber dependence in the Neo-fiber law allows a more accurate approximation to physiological cardiac motion, as the lower average and maximum errors indicate that the Neo-fiber can on average reproduce most of the deformation modes considered. The approximation to cardiac motion is further improved as the exponent $\alpha$ increases. Finally, the reduced form of the Holzapfel–Ogden law presents the smallest average and minimum error between the deformations produced using a given constitutive law and the Guccione law, confirming that the values used for the exponents $b$ and $b_f$ are appropriate and allow for physiological cardiac deformation. This is also illustrated in the meshes in Fig. 11 which present the maximum difference from Guccione combinations. For the Neo-fiber and reduced Holzapfel–Ogden models, even the maximum difference from the ground truth is still quite small as indicated by the close match between model and ground truth cardiac deformation.

**Fig. 12:**
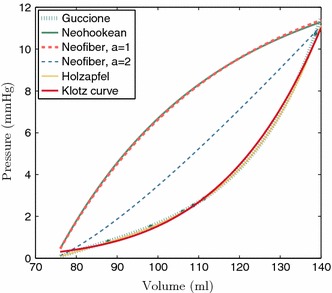
Typical end-diastolic pressure–volume curves for the constitutive laws considered and ground truth Klotz curve. While the reduced Holzapfel–Ogden and Guccione laws are able to reproduce the Klotz curve, the Neo-Hookean and Neo-fiber laws cannot produce a physiological pressure–volume response

### Comparison of models’ EDPVRs

End-diastolic pressure–volume relation (EDPVR) is an important determinant of cardiac function; therefore, the considered constitutive laws were compared with respect to their ability to reproduce a physiological EDPVR. For this comparison, the empirical EDPVR proposed by Klotz et al. (2006) was chosen as the ground truth physiological EDPVR, as it is considered capable to represent healthy and diseased cases. The EDPVR is derived from a single set of end-diastolic pressure (EDP) and volume (EDV) measurements, which for the in silico tests were chosen as EDP = 11 mmHg, EDV = 140  ml.

Figure 12 illustrates typical EDPVRs for the various constitutive laws considered. As indicated by the curve, the Neo-Hookean and Neo-fiber laws were not able to reproduce a physiological EDPVR, even though case $a=2$ gives a better approximation for the Neo-fiber law. On the contrary, the exponential Guccione and the reduced Holzapfel–Ogden laws were able to provide a physiological EDPVR as indicated by the close match to the Klotz curve.

### In vivo behavior of $\mathcal {J}$

The in silico tests performed in Sects. 3.1, 3.2 and 3.3 suggest that the reduced Holzapfel–Ogden law combines practical identifiability with good representation of cardiac deformation and EDPVR. Therefore, the reduced Holzapfel–Ogden is suitable for patient-specific applications as it is an accurate cardiac model with reliable—thus potentially clinically important parameters.

In this section the reduced Holzapfel–Ogden law is employed in an in vivo case of a healthy volunteer. The behavior of the objective function $\mathcal {J}$ is examined in this setting as well, to allow for the characterization of practical identifiability in a real-world scenario. Within this setting, we can also assess the effect of model fidelity on the parameter estimation process, as the simulations are now compared with actual cardiac deformation data.

The extracted 3D displacement field was compared with passive inflation simulation results to provide parameter estimates for the reduced Holzapfel–Ogden law. As the LV pressure trace was not part of the available data, we were not able to obtain unique values for each parameter separately. However, taking advantage of the linear dependence of the law on the parameters and accordingly their proportionality to inflating pressure, we were able to uniquely estimate the ratio $\alpha _f /\alpha$, which is independent of the inflating pressure. Note that if the end-diastolic pressure is available, we can retrieve the actual values of $\alpha _f$ and $\alpha$ by multiplying by the ratio between the actual pressure values and the values used in the estimation process.

**Fig. 13:**
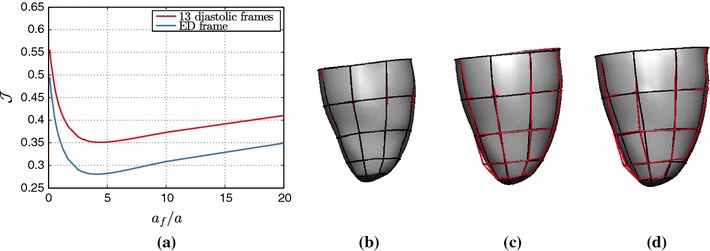
**a** $\mathcal {J}$ over the ratio $\alpha _f / \alpha$ for the reduced Holzapfel–Ogden law, when 13 or only the final diastolic frames are considered within $\mathcal {J}$. The match between the model (volume and *black lines*) and data (*red lines*) at **b** early-, **c** mid- and **d** end-diastole is illustrated in a simulation with the obtained minimum ratio

Figure 13 illustrates the behavior of $\mathcal {J}$ over a range of ratios $\alpha _f /\alpha$, where the fiber parameter $\alpha _f$ was kept constant. Even though in this in vivo case we cannot assess the error in the parameter estimates, we can still infer that the practical identifiability of the reduced Holzapfel–Ogden law observed in in silico tests 3.1.3 is maintained when actual 3D tags are used, based on the distinct and unique minimum. The identifiability of the law using all or only the final diastolic frames is examined as well, indicating that $\mathcal {J}$ presents distinct and unique minima in both cases, with a 12  % difference between the two estimated values.

The similarity in behavior of $\mathcal {J}$ over the parameter ratio between in silico and in vivo tests (see Figs. 9, 13) confirms model validity for the reduced Holzapfel–Ogden law. Finally, it offers a validation of our in silico testing protocol, thus allowing for the conclusions for the considered constitutive laws to be extrapolated to parameter estimation applications with real 3D tags.

### Study limitations

A relatively coarse, lower order mesh was used for the in silico example. This choice was based on the small computational time per simulation, which allowed for the large number of simulations performed to provide the landscape of the objective function over the parameter space. Nevertheless, as illustrated in Appendix 2, the mesh resolution used was sufficient to examine parameter identifiability characteristics in our models, for both the in silico and in vivo tests.

Further, only one objective function has been considered, even though other objective functions might be able to elucidate other characteristics for the behavior of the various laws. Other objective functions are not considered here, as such a study would require proving that a potential objective function is discerning. If non-discerning, proving that unique parameter identification is achievable becomes challenging as it then must rely on the observations not exposing non-uniqueness of the objective function. Nevertheless, the chosen $\mathcal {J}$ uses an $\varvec{L}^2$ norm on the displacements, which is generally considered a robust criterion and should be able to provide adequate information and accurately describe the identifiability characteristics of each law.

Ten diastolic frames were used as observations in the in silico test. This choice was based on the number of diastolic frames in the available 3D tagged MRI data (e.g., where the cavity volume is increasing). However, the number of diastolic image frames used in other studies is variable with authors considering all or part of diastole. This variability is due to assumptions on residual active tension, the presence of which is confirmed by decreasing cavity pressures even after the opening of the mitral valve (Pasipoularides et al. 1986). As a result, early diastolic frames do not contain purely passive tissue behavior, but also contain residual active stress. We observed that parameter identifiability for the reduced Holzapfel–Ogden model was preserved in both in silico and in vivo scenarios, using only the last diastolic frame (see Figs. 9 and 13). $\mathcal {J}$ presented unique and distinct minima for all cases, with a 12 % difference between the two estimated values in the in vivo case. However, this robustness is likely due to the single parameter ($\alpha _f/\alpha$ ratio) estimated over the entire left ventricle. Incorporation of spatially varying parameters would increase sensitivity to noise and would likely require additional passive diastolic frames to ensure identifiability.

Even though the effect of unbiased noise is examined, several aspects of the process or the data that may have a biased influence are not considered. For instance, the resolution and number of tagged lines in the data along with the tracking algorithm used are likely to incorporate consistent error in the parameter estimation process. The boundary conditions used in the simulations are also likely to influence the identifiability and model fidelity results. Understanding these attributes is important for patient-specific personalization; therefore, further work is required to clarify the influence of these factors on the landscape of the objective function and the estimation process in general.

The reference configuration is assumed to be known and correspond to a specific frame of diastole in the in vivo example. Even though this is a common approach, we have verified that the identifiability of model parameters is not sensitive to the frame used as a reference configuration for the in silico case (see Appendix 3). Interestingly, the parameter ratio is relatively consistent (20 % maximum error between actual and estimated ratio) irrespective of the reference domain. We note, however, that this might be an artifact of the idealized geometry used or other simplifications inherent in the model produced data.

Finally, only one in vivo case is considered; thus, further work is needed to examine effects in vivo, such as noisy or low-resolution data, or diseased cases where the cardiac deformation is likely to differ significantly. However, the in silico tests performed provide a standard, giving the “best case scenario,” which can be anticipated when using real data. Further, the in vivo and in silico model fidelity of the reduced Holzapfel–Ogden law encourage the application of the law to diseased cases as well.

## Conclusions

In this paper, we examine the practical identifiability and model fidelity of a range of cardiac constitutive laws using 3D tagged MRI as the available data. In order to investigate the practical identifiability of the laws considered and examine the potential of using 3D tags in parameter estimation applications, we generate synthetic 3D tags directly from simulation results and assess the behavior of the objective function over the parameter space through parameter sweeps. The laws considered are also compared with respect to their ability to represent physiological cardiac motion and EDPVR, elucidating the primary components that should guide the choice of an appropriate cardiac constitutive law—namely reliable parameters and adequate representation of cardiac deformation and function.

Our results verify the reported coupling of the transversely isotropic Guccione law, suggesting the need for a law with better identifiability characteristics that would allow for reliable parameter estimates. The Neo-Hookean law is shown to have good identifiability characteristics, due to linear parameter dependence. The stiffness parameter is identifiable, provided adequate deformation is present in the available data. However, due to its isotropy, Neo-Hookean deformation misses key characteristic deformation modes, mainly long-axis elongation and twist. Further, it cannot reproduce physiological pressure–volume response.

Building on the Neo-Hookean model, the Neo-fiber law maintains the good identifiability characteristics, while reproducing physiological cardiac deformation. Both parameters are identifiable, even though sufficient deformation is required to allow identifiability of the fiber parameter due to the structure of the constitutive law. However, using the Neo-fiber law leads to inaccurate pressure–volume response, which cannot match the empirical Klotz curve.

The reduced Holzapfel–Ogden law, however, combines all important attributes considered, offering a balance between practical identifiability and adequate representation of cardiac deformation and EDPVR. Its use in an in vivo case where good identifiability characteristics are maintained supports the conclusion that the reduced Holzapfel–Ogden law offers a sensible choice in patient-specific applications with 3D tagged data.

## Figures and Tables

**Fig. 14:**
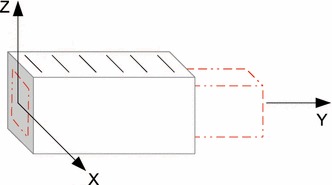
The block of tissue under consideration. The *red lines* show the deformed configuration

**Fig. 15:**
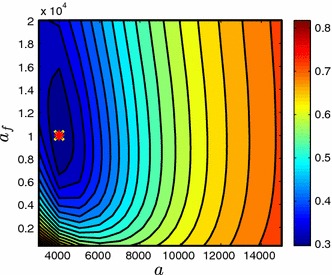
Landscape of objective function $\mathcal {J}$ of reduced Holzapfel–Ogden law over parameter space, when a finer mesh consisting of 448 elements is used

**Fig. 16:**
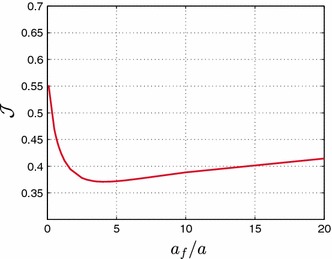
$\mathcal {J}$ over the ratio $\alpha _f/\alpha$ for the reduced Holzapfel–Ogden law in vivo, when a finer mesh consisting of 576 elements is used

**Fig. 17:**
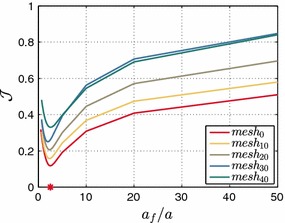
$\mathcal {J}$ over the parameter ratio $\alpha _f/\alpha$ for the reduced Holzapfel–Ogden law. Five different reference configurations are used, which correspond to different diastolic phases

## Appendix 1: Structural identifiability for cardiac laws

In this section, we examine an application of Theorem 2, where we study the structural identifiability of the laws considered. Specifically, we consider a block of tissue (Fig. 14) and show that for the Neohookean, Neo-fiber and reduced Holzapfel–Ogden laws, which have a linear dependence on their parameters, a single pure tension experiment is sufficient to prove the bijectivity of their $\varphi$ mapping, thus ensuring their structural identifiability.

We can consider a block of incompressible tissue (Fig. 14), under pure tension in one of the three directions. The body is fully constrained at $(0, 0, 0)$ and is under the influence of zero traction on the side boundaries.

For the case of the Neohookean law, due to its isotropy, pure tension in any of the three directions is sufficient to ensure structural identifiability. Specifically, for elongation in the $X$ direction the deformation gradient $\varvec{F}$ and left Cauchy-Green deformation tensor can be expressed as

24 $$\begin{aligned} \varvec{F}= \left[ \begin{array}{c@{\quad }c@{\quad }c} \lambda &{} 0 &{} 0 \\ 0&{} \frac{1}{\sqrt{\lambda }} &{} 0 \\ 0 &{} 0 &{} \frac{1}{\sqrt{\lambda }} \end{array} \right] , \quad \varvec{b}= \left[ \begin{array}{c@{\quad }c@{\quad }c} \lambda ^2 &{} 0 &{} 0 \\ 0&{} \frac{1}{\lambda } &{} 0 \\ 0 &{} 0 &{} \frac{1}{\lambda } \end{array} \right] \end{aligned}$$

where $\lambda$ denotes the stretch of the body in the $X$ direction, and the deformation in the $Y$ and $Z$ components is derived from the symmetry and incompressibility of the body. As the Neohookean law has only one parameter, expression 13 becomes $\varvec{\sigma }= \mu \varvec{\sigma }_1$, where using definition 4 and $J=1$,

25 $$\begin{aligned} \varvec{\sigma }_1= \left( \lambda ^2- \frac{1}{\lambda }\right) \left[ \begin{array}{c@{\quad }c@{\quad }c} \frac{2}{3} &{} 0 &{} 0 \\ 0&{} -\frac{1}{3} &{} 0 \\ 0 &{} 0 &{}- \frac{1}{3} \end{array} \right] . \end{aligned}$$

If we choose the test function $\varvec{v}=\big [\frac{1}{2}\varvec{x}, -\frac{1}{4}\varvec{y}, -\frac{1}{4}\varvec{z}\big ]$, $\varvec{A}$ in Theorem 2 is now a nonzero scalar [$\varvec{A}= \frac{1}{2}(\lambda ^2- \frac{1}{\lambda })$], for any nonzero elongation ($\lambda \ne 1$). This ensures that $\phi$ is bijective, suggesting structural identifiability of the Neohookean law.

The Neo-fiber and reduced Holzapfel–Ogden law can also be shown to be structurally identifiable through a pure tension experiment, where the elongation is exerted in the cross-fiber direction $Y=0$ (note that for elongation in the fiber direction, the matrix $\varvec{A}$ becomes singular for any choice of test functions $\varvec{v} \in \varvec{W}_{0,Div}^u$). The deformation gradient and left Cauchy-Green tensor in this case are described by

26 $$\begin{aligned} \varvec{F}= \left[ \begin{array}{c@{\quad }c@{\quad }c} \frac{1}{\sqrt{\lambda }} &{} 0 &{} 0 \\ 0&{} \lambda &{} 0 \\ 0 &{} 0 &{} \frac{1}{\sqrt{\lambda }} \end{array} \right] , \quad \varvec{b}= \left[ \begin{array}{c@{\quad }c@{\quad }c} \frac{1}{\lambda } &{} 0 &{} 0 \\ 0&{} \lambda ^2 &{} 0 \\ 0 &{} 0 &{} \frac{1}{\lambda } \end{array} \right] \end{aligned}$$

where $\lambda$ represents stretch in the cross-fiber direction, and $\varvec{{f}_0}=[1 \ 0 \ 0 ]$. According to 13, the stress tensors for the Neo-fiber and reduced Holzapfel–Ogden law can be written as $\varvec{\sigma }= C_2 \varvec{\sigma }_1 + C_1' \varvec{\sigma }_2$ and $\varvec{\sigma }= \alpha \varvec{\sigma }_1 + 2\alpha _f \varvec{\sigma }_2$, respectively, where for simplicity $C_1'= C_1-C_2$. Taking the specific deformation mode into account, $\varvec{{f}} = [ \frac{1}{\sqrt{\lambda }} \ 0 \ 0]$, $I_{\hat{C}_f}= \frac{1}{\lambda }$ and the two stress components for the Neo-fiber law are

27 $$\begin{aligned} \varvec{\sigma }_1&= \left( \lambda ^2- \frac{1}{\lambda }\right) \left[ \begin{array}{c@{\quad }c@{\quad }c} -\frac{1}{3} &{} 0 &{} 0 \\ 0&{} \frac{2}{3} &{} 0 \\ 0 &{} 0 &{}- \frac{1}{3} \end{array} \right] \end{aligned}$$

28 $$\begin{aligned} \varvec{\sigma }_2&= \frac{1}{\lambda }\left( \frac{1}{\lambda }-1\right) ^{\alpha }\left[ \begin{array}{c@{\quad }c@{\quad }c} \frac{2}{3} &{} 0 &{} 0 \\ 0&{} -\frac{1}{3} &{} 0 \\ 0 &{} 0 &{}- \frac{1}{3} \end{array} \right] . \end{aligned}$$

Similarly, the two stress component for the reduced Holzapfel–Ogden law can be expressed as

29 $$\begin{aligned} \varvec{\sigma }_1&= \exp \left[ 5\left( \lambda ^2+\frac{2}{\lambda }-3\right) \right] \left( \lambda ^2- \frac{1}{\lambda }\right) \left[ \begin{array}{c@{\quad }c@{\quad }c} -\frac{1}{3} &{} 0 &{} 0 \\ 0&{} \frac{2}{3} &{} 0 \\ 0 &{} 0 &{}- \frac{1}{3} \end{array} \right] \end{aligned}$$

30 $$\begin{aligned} \varvec{\sigma }_2&= \exp \left[ 5\left( \frac{1}{\lambda }-1\right) ^2\right] \frac{1}{\lambda }\left( \frac{1}{\lambda }-1\right) \left[ \begin{array}{c@{\quad }c@{\quad }c} \frac{2}{3} &{} 0 &{} 0 \\ 0&{} -\frac{1}{3} &{} 0 \\ 0 &{} 0 &{}- \frac{1}{3} \end{array} \right] .\nonumber \\ \end{aligned}$$

Clearly, the two components of the Neo-fiber and reduced Holzapfel–Ogden law have the same matrix structure, which can be represented as

31 $$\begin{aligned} \varvec{\sigma }_1= \left[ \begin{array}{c@{\quad }c@{\quad }c} \alpha _1 &{} 0 &{} 0 \\ 0&{} \beta _1 &{} 0 \\ 0 &{} 0 &{} \alpha _1 \end{array} \right] , \quad \varvec{\sigma }_2= \left[ \begin{array}{c@{\quad }c@{\quad }c} \alpha _2 &{} 0 &{} 0 \\ 0&{} \beta _2 &{} 0 \\ 0 &{} 0 &{} \beta _2 \end{array} \right] \end{aligned}$$

If we then choose our test functions to be $\varvec{v_1}= \big [\frac{1}{2}\varvec{x}, -\frac{1}{4}\varvec{y}, -\frac{1}{4}\varvec{z}\big ]$ and $\varvec{v_2}= \big [-\frac{1}{4}\varvec{x}, \frac{1}{2}\varvec{y}, -\frac{1}{4}\varvec{z}\big ]$, matrix $\varvec{A}$ in Theorem 2 becomes

32 $$\begin{aligned} {\varvec{A}}= \left[ \begin{array}{c@{\quad }c} \frac{1}{4}(\alpha _1- \beta _1 ) &{} \frac{1}{2}(\alpha _2- \beta _2) \\ \frac{1}{2}(\beta _1- \alpha _1 )&{} \frac{1}{4}(\beta _2- \alpha _2 ) \end{array} \right] , \end{aligned}$$

whose determinant $|\varvec{A}|= \frac{3}{16}(\alpha _1- \beta _1)(\alpha _2- \beta _2)$ is nonzero due to the structure of $\varvec{\sigma }_1$ and $\varvec{\sigma }_2$. Based on Theorem 2, the invertibility of $\varvec{A}$ ensures the bijectivity of $\varphi$ for the Neo-fiber and reduced Holzapfel–Ogden laws, proving that they are structurally identifiable.

## Appendix 2: Effect of mesh resolution on $\mathcal {J}$ behavior

In order to investigate the effect of the mesh resolution on the behavior of the objective function, some of the tests presented are repeated on a finer mesh. Specifically, we have repeated the in silico characterization of the reduced Holzapfel–Ogden law presented in Sect. 3.1.3 on a uniformly refined mesh (448 elements, instead of 56 elements used in the initial test). As can be deduced by the similarity in both the landscape of $\mathcal {J}$ and the estimated values between Figs. 8 and 15, the coarser mesh used for the in silico tests is able to characterize the identifiability of the considered constitutive laws.

Similarly, the in vivo test described in Sect. 3.4 was repeated on a finer mesh consisting of 576 elements instead of 72 elements used in the initial test. Comparing Figs. 13 and 16, we can deduce that the behavior of $\mathcal {J}$ is consistent between the two meshes, suggesting that we can rely on the conclusions deduced from the use of the coarser mesh. We note that the small difference in the obtained ratio in the in vivo test could be justified by the fact the finer mesh was also used to propagate the extracted myocardial motion, and its cavity volume was used to drive the simulation.

## Appendix 3: Effect of reference configuration on $\mathcal {J}$ behavior

This section investigates the effect of choosing a different diastolic frame as a reference configuration on the behavior of the objective function. Specifically, we have used an in silico test to examine the effect of the reference configuration on the identifiability of the parameter ratio of the reduced Holzapfel-Ogden law, in a volume-prescribed diastolic filling simulation. Five different reference configurations were considered for the simulations performed over parameter sweeps: the initial reference mesh and meshes corresponding to loading steps 10, 20, 30 and 40 of the simulation used for the creation of the synthetic tags. In each case, the number of loading steps and thus observations was adjusted, so that each observation would correspond to 5 loading steps.

Figure 17 compares the behavior of the objective function $\mathcal {J}$ when different diastolic phases are used as the reference configuration. Based on the similar behavior of the curves, we can deduce that the identifiability of the parameter ratio is not affected by the assumed reference configuration.

## Footnotes

1
http://www.doc.ic.ac.uk/~dr/software.
